# Dysregulated glucocorticoid-responsive immune genes in peripheral blood mononuclear cells as a shared molecular signature of autism spectrum disorder and irritable bowel syndrome

**DOI:** 10.1371/journal.pone.0353181

**Published:** 2026-07-09

**Authors:** Kuo Zhang, Fangfang Mou, Jing Liu, Jianzhong Wang

**Affiliations:** Qinhuangdao Maternity and Child Health Hospital, Qinhuangdao, Hebei, China; Taipei Veterans General Hospital, TAIWAN

## Abstract

**Background:**

Autism spectrum disorder (ASD) is frequently accompanied by gastrointestinal (GI) disturbances resembling irritable bowel syndrome (IBS). While dysregulation of the hypothalamic–pituitary–adrenal (HPA) axis and impaired glucocorticoid-responsive immune (GRI) signaling are proposed links between these disorders, the precise molecular mechanisms remain poorly understood.

**Methods:**

We performed an integrative transcriptomic analysis of peripheral blood mononuclear cells (PBMCs) from ASD and IBS cohorts. Our approach combined single-sample Gene Set Enrichment Analysis (ssGSEA), differential expression profiling, weighted gene co-expression network analysis (WGCNA), and machine-learning-based feature selection. We utilized single-cell RNA sequencing to resolve cellular sources, while transcription factor, miRNA, and Connectivity Map (CMap) analyses identified regulatory mechanisms and potential drug candidates for reversing GRI-associated signatures.

**Results:**

GRI-associated transcriptional activity was markedly elevated in the ASD group and moderately upregulated in the IBS group. Network and enrichment analyses revealed a convergence of immune recognition and cytokine signaling pathways. We identified four core genes—LRFN1*,* NUAK2*,* TMEM154*,* and GAPT—that consistently discriminated disease status. These genes were primarily expressed in monocytes, natural killer (NK) cells, and B cells. Regulatory analysis implicated stress-responsive transcriptional control and extensive miRNA modulation in these processes. CMap analysis identified RN-486, saracatinib, and batimastat as compounds predicted to restore GRI homeostasis.

**Conclusions:**

These findings define a shared GRI-associated molecular signature linking systemic stress adaptation to immune dysregulation along the brain–gut axis. This study provides novel mechanistic insights and identifies potential transcriptomic biomarkers and therapeutic targets addressing the shared molecular architecture between ASD and IBS.

## Introduction

Autism spectrum disorder (ASD) is a complex and heterogeneous neurodevelopmental disorder characterized by persistent impairments in social communication and the presence of restricted repetitive behaviors [1]. Global epidemiological data reveal significant variability in ASD prevalence, with reported rates ranging from approximately 0.01% to as high as 4.36% across different populations. These discrepancies are largely attributed to international variations in diagnostic infrastructure, socioeconomic factors, and the evolving sensitivity of screening tools. Despite these regional differences, the consistently observed prevalence underscores the growing global burden of the disorder and its profound societal and clinical impact [2]. Mounting evidence suggests that its origins lie in a dynamic interplay between strong genetic predisposition and a spectrum of environmental and developmental influences that perturb the maturation and functional integration of neural systems [3]. Given that ASD is a broad spectrum, such inherent clinical heterogeneity underscores the necessity of identifying robust, shared molecular signatures that transcend diverse demographic and diagnostic backgrounds.

Although ASD has conventionally been conceptualized as a disorder of brain development, accumulating evidence indicates that its effects extend systemically to include physiological abnormalities beyond the central nervous system [4]. Gastrointestinal (GI) disturbances are particularly prevalent, with many individuals exhibiting symptoms consistent with irritable bowel syndrome (IBS) [5]. Epidemiological and genetic studies highlight a robust link between ASD and IBS. A large-scale study of 35,048 individuals found that higher autistic scores significantly associate with increased IBS risk (OR = 1.44, 95% CI: 1.34–1.55) [6]. This clinical association is further supported by genetic evidence showing a significant shared genetic architecture (r_g_ = 0.27, adjusted p = 2.04 × 10^−7^) between the two conditions [5]. Such clinical convergence suggests that both disorders may share common biological roots that couple the neural, immune, and enteric systems along the gut–brain axis.

IBS is a chronic, stress-sensitive functional disorder defined by recurrent abdominal pain and altered bowel habits in the absence of structural pathology [7]. It is now regarded as a prototypical disorder of gut–brain interaction, driven by dysregulated communication among central stress circuits, immune signaling, and the enteric nervous system [8]. Given that many physiological perturbations in IBS mirror those in ASD and are similarly disrupted by stress-related abnormalities, a compelling hypothesis posits that HPA axis dysfunction and impaired glucocorticoid (GC) signaling represent a shared pathophysiological link between the two disorders [9,10]. The HPA axis orchestrates the organism’s response to stress, with glucocorticoids released from the adrenal cortex acting on immune cells to impose transcriptional programs that restrain inflammation and restore homeostasis [11]. Perturbations in this regulatory loop, manifesting as altered cortisol rhythms, impaired receptor sensitivity, or maladaptive expression of GC-responsive immune (GRI) genes, can sustain a chronic low-grade inflammatory milieu [9,10]. Dysregulated HPA/GC signaling has been documented in both ASD (characterized by blunted or irregular cortisol responses) [10,12,13] and IBS (marked by exaggerated or prolonged stress reactivity) [14,15], implicating impaired GC-mediated immune regulation as a biological bridge that unites neural and peripheral dysfunctions under these conditions. Although GRI-associated genes are strongly implicated in both ASD and IBS, Ferguson et al. further link stress-responsive biomarkers to behavioral symptoms in ASD, suggesting that heightened stress reactivity in individuals with GI symptoms was observed without concurrent significant changes in stress-associated cytokine concentrations, which warrants deeper investigation [16].

Peripheral blood mononuclear cells (PBMCs) offer a minimally invasive window into systemic physiology, capturing the interplay between immune activity, neuroendocrine signaling, and stress responses in ASD [17,18]. As a heterogeneous population of lymphocytes and monocytes, PBMCs are particularly responsive to GC signals and inflammatory cues, making them a sensitive readout of the HPA axis and GRI dysregulation [19]. Moreover, PBMCs reflect peripheral signals linked to gut-brain-immune interactions, providing a practical proxy for investigating the shared molecular basis between ASD and IBS [20]. Integrating bulk- and single-cell transcriptomics from PBMC with network and predictive analytics enables the identification of shared transcriptional programs and mechanistic pathways underlying these intersecting disorders.

In this study, we tested the hypothesis that ASD and IBS share a reproducible and dysregulated GRI signature detectable in PBMC transcriptomes. To this end, we initially applied single-sample gene set enrichment analysis (ssGSEA) to reduce the dimensionality of the GRI gene set and to quantify its activity in individual samples. Integrative analyses combining differential expression, pathway enrichment, and weighted gene co-expression network analysis (WGCNA) were performed to identify concordant modules and their associations with GRI scores, followed by machine-learning-based feature selection to define a compact core gene signature. Single-cell RNA (scRNA) sequencing was subsequently used to localize this signature to specific immune populations, while transcription factor motif and miRNA analyses were applied to infer up- and downstream regulatory drivers, respectively. Finally, to evaluate translational potential, we leveraged the Connectivity Map (CMap) resource to identify repurposable compounds predicted to reverse the GRI-associated transcriptional pattern simultaneously in ASD and IBS.

By uniting transcriptomic studies with mechanistic inference and perturbational mapping, this study aimed to (1) define a validated PBMC GRI-related signature common to ASD and IBS, (2) delineate its cellular and regulatory origins, and (3) identify candidate therapeutics capable of restoring GRI homeostasis. Collectively, these efforts seek to illuminate a mechanistic bridge between the brain and gut in two clinically burdensome disorders, and to provide actionable leads for biomarker discovery and therapeutic repurposing.

## Methods

### Data collection and pre-processing

We obtained bulk transcriptomic datasets of PBMCs for IBS and ASD from public repositories (Table 1). The analysis included two independent cohorts for each condition. Within each independent dataset, cases and controls were meticulously checked to minimize demographic confounders. Notably, the GSE124549 dataset was profiled using a custom NanoString panel that targets glucocorticoid-responsive immune (GRI) genes. To ensure high cohort comparability, we employed a parallel-and-intersection analysis framework rather than direct amalgamation of cross-disease data. Specifically, data integration and batch correction using the *SVA* R package were performed within each disease entity. Then, data were normalized with the “normalizeBetweenArrays” function from the *limma* R package [21,22]. We annotated gene probes with official gene symbols and retained the most highly expressed probes for genes mapped using multiple probes. To further validate the robustness of the identified signature, we deliberately integrated a medication-free single-cell RNA-seq dataset (GSE217850 from South Korea) of ASD PBMCs with sex- and age-matched controls (GSM6616992, GSM6616995) sourced from GSE214865. This can ensure that the results of core molecular feature remain consistent in a more controlled biological context. All single-cell data were generated using an Illumina NovaSeq 6000 platform (GPL24676).

**Table 1:** Characteristics of collected PBMC expression datasets for IBS and ASD.

No.	Disease	Database source	Accession number	Platform	Tissue	Healthy control (n)	Patients (n)
1	IBS	GEO	GSE124549	GPL25996	PBMC	66	31
2	IBS	GEO	GSE63379	GPL17586	PBMC	32	35
3	ASD	GEO	GSE77103	GPL17077	PBMC	4	4
4	ASD	ArrayExpress	E-MTAB-13871	GPL24676	PBMC	13	12

GEO: Gene Expression Omnibus (https://www.ncbi.nlm.nih.gov/geo/);

ArrayExpress (https://www.ebi.ac.uk/biostudies/arrayexpress);

Datasets were sourced from Japan (GSE77103), Italy (E-MTAB-13871), USA (GSE124549 and GSE63379) to ensure demographic diversity.

### Differentially expressed gene and enrichment analysis

Differentially expressed gene (DEG) analysis of ASD and IBS datasets was performed using the *limma* R package. Genes with an absolute log2 fold change (|logFC|) > 0 and a p-value < 0.05 were considered statistically significant and retained for subsequent analysis. Data visualizations were generated using the SRPlot online server (https://www.bioinformatics.com.cn/). We then defined the DEGs identified from the GSE124549 dataset (an IBS cohort profiled for GRI genes) as a custom signature gene set for ssGSEA evaluation. ssGSEA was performed using the *GSVA* R package [23]. Statistical significance for group comparisons of ssGSEA enrichment scores was evaluated using unpaired two-tailed Student’s *t*-tes*t*s conducted via GraphPad Prism 8.3.0.

### WGCNA analysis

We conducted a WGCNA analysis using the *WGCNA* R package to identify gene modules associated with clinical traits [24]. After pre-processing, the 5,000 most variable genes, determined by standard deviation, were selected for network construction. To detect and remove outliers, hierarchical clustering was performed using the average linkage method. Soft-thresholding power was determined through network topology analysis to ensure a scale-free topology (R² > 0.9). An adjacency matrix was then constructed and transformed into a topological overlap matrix (TOM) to quantify network connectivity. Gene modules were defined using dynamic tree cutting, with a minimum module size of 30 genes. Modules with highly correlated eigengenes (correlation > 0.7) were merged by using a cut height of 0.3. Module–trait associations were evaluated by computing Pearson correlations between module eigengenes and clinical traits, including GRI ssGSEA enrichment scores and disease status. Statistical significance was determined using the Student’s asymptotic *p*-value. For modules of particular interest, hub genes were identified based on two criteria: module membership (MM), the correlation between a gene’s expression and its module eigengene; and gene significance (GS), the absolute correlation between gene expression and clinical traits. Hub genes were defined as |MM| > 0.5, and |GS| > 0.5. Relationships between module preservation and gene significance were visualized using custom R scripts.

### Protein-protein interaction network construction

Protein-protein interaction (PPI) data were retrieved from the STRING database (version 12.0; https://string-db.org) for *Homo sapiens*, applying a confidence score threshold of 0.4 to define significant interactions. The resulting network was imported into the Cytoscape software (version 3.9.1) for visualization and analysis, where nodes represent proteins and edges represent associations. We then performed topological analysis using the built-in “Analyze Network” function to calculate the node degree and identify highly connected proteins as potential hub genes. Isolated nodes without connections were removed from the final network visualization to enhance clarity.

### Gene ontology and kyoto encyclopedia of genes and genomes pathway enrichment analysis

Gene Ontology (GO) and Kyoto Encyclopedia of Genes and Genomes (KEGG) pathway enrichment analysis were performed using the *clusterProfiler* and *org.Hs.eg.db* R packages [25,26]. To focus on biologically relevant pathways, KEGG terms associated with cancer and other disease categories that were not directly related to IBS or ASD pathogenesis were excluded from the results. Enrichment results were visualized using the SRplot online platform, with terms arranged in descending order based on their log_10_ (p-value).

### Machine learning framework for feature selection

First, the SVA-integrated disease-specific dataset was randomly split into a training set (70%) and an independent test set (30%) using the “createDataPartition” function in the *caret* R package, ensuring proportional class representation in both subsets. Subsequently, we implemented a comprehensive machine learning framework with eight distinct algorithms. Such consensus across multiple machine learning algorithms was employed as a robustness-oriented strategy to identify molecular signatures that remained stable across different computational frameworks rather than relying on the performance of a single predictive model. Each model was trained and optimized using 5-fold cross-validation on the training set, and the final performance was evaluated on the held-out test set based on accuracy, area under the receiver operating characteristic curve (AUC-ROC), sensitivity, and specificity. To reduce the risk of overfitting associated with the modest sample size, all feature selection and hyperparameter optimization procedures were performed exclusively within the training set, while the independent test set was reserved solely for final model evaluation. Finally, feature importance was quantified using model-specific criteria: coefficients for Least Absolute Shrinkage and Selection Operator (LASSO) and Generalized Linear Model (GLM), Gini importance for Random Forest, feature importance values for eXtreme Gradient Boosting (XGBoost), and built-in variable importance functions in *caret* for the remaining models. The detailed procedure for each algorithm is as follows:

(1) LASSO: We fitted a binomial model with an L1 penalty using the *glmnet* R package [27]. The optimal regularization parameter (*λ*) was determined based on the minimum deviance. Features with nonzero coefficients at λ.1se were selected.
(2) SVM-RFE (Support Vector Machine–Recursive Feature Elimination): Recursive feature elimination was performed using the “rfe” function in *caret*, with a radial basis function SVM as the base classifier. Feature subsets ranging from 5 to 120 were evaluated, and the subset that yielded the lowest cross-validation error was chosen.
(3) Random Forest: We constructed a forest of 500 trees using the *randomForest* R package [28]. The “mtry” parameter (number of features randomly sampled at each split) was tuned from 2 to 50, for the value that minimized the out-of-bag (OOB) error. Feature importance was quantified using the mean decrease in Gini.
(4) XGBoost: Using the *xgboost* R package, we optimized the hyperparameters (*max_depth, eta, gamma, colsample_bytree, min_child_weight,* and *subsample*) via a random search in *caret*, utilizing AUC as the performance metric [29]. The final model was retrained using the optimal parameters.
(5) ANN (Artificial Neural Network): A single-hidden-layer feedforward network was trained using the *nnet* R package within the *caret* framework [30]. The model was tuned for the number of hidden units and weight decay using the top 20% of genes with the highest variance as input features.
(6) GLM: We fitted a regularized binomial logistic regression model using the *glmnet* R package, and the elastic-net mixing parameter (α) and regularization strength (λ) were optimized through cross-validation using the same high-variance gene set as in the ANN model.
(7) K-Nearest Neighbors (KNN): Feature selection and model training were implemented using the “rfe” function in *caret*. The optimal number of neighbors (*k*, ranging from 1 to 20) was determined via cross-validation.
(8) Naive Bayes (NB): Implemented with the *klaR* R package via *caret*, the model was trained on the top 20% of high-variance genes, with hyperparameters (*usekernel*, *fL*, and *adjust*) optimized for the best performance [31].

### scRNA sequencing analysis

scRNA sequencing data were processed and analyzed in R using the *Seurat* (v5.0.0) package [32]. Initially, low-quality cells were removed based on two criteria: cells expressing fewer than 200 genes, and genes detected in fewer than three cells. Subsequently, the dataset underwent standard *Seurat* pre-processing, including normalization, identification of highly variable features, scaling, and principal component analysis (PCA). To identify and exclude potential doublets, *DoubletFinder* was applied to the pre-processed Seurat object using dataset-optimized parameters, and the predicted doublets were removed from downstream analyses [33]. For datasets derived from multiple samples or batches, data integration was performed using *Seurat*’s reciprocal PCA (RPCA) workflow. Integration anchors were identified using the “FindIntegrationAnchors” function, and datasets were integrated using “IntegrateData,” effectively mitigating batch effects. Following integration, cell-type annotation was performed using the *SingleR* R package with the MonacoImmuneData reference, which provides well-characterized transcriptomic signatures of major human immune cell types [34]. Finally, marker genes for each annotated cell subtype were identified using the FindAllMarkers function, with thresholds set at |avg_log₂FC| > 1, adjusted *p*-value (p_val_adj) < 0.05, and min. pct > 0.1. Genes meeting all three criteria were considered to be statistically significant.

### Transcription factor motif enrichment analysis

We performed transcription factor (TF) motif enrichment analysis using the *RcisTarget* R package (v1.24.0) [35]. Motif enrichment analysis was conducted using the hg19–500 bp-upstream-7species.mc9nr.genes_vs_motifs.rankings.feather database with derived Catalog of Inferred Sequence Binding Preferences (CIS-BP) motifs for annotation. Key analysis parameters included a normalized enrichment score (NES) threshold of 3.0, an AUC maximum rank threshold set to 3% of the total motifs, and a gene enrichment maximum rank of 5000 genes. High-confidence annotations include direct and orthology-based inferences. Following the enrichment analysis, significant genes associated with each motif were identified, and motif logos were incorporated for visualization. The results were validated through a recovery curve analysis of top-ranking motifs and presented in both interactive and static formats for comprehensive evaluation.

### miRNA-target interaction analysis

miRNA-target interactions were predicted using the starBase database (https://rnasysu.com/encori/index.php) to investigate potential post-transcriptional regulation of hub genes by miRNAs. Putative miRNA-mRNA pairs were identified with stringent thresholds: a TDMDScore > 0.8 to select for interactions potentially involved in target-directed miRNA degradation, a phyloP score > 0.6 to ensure evolutionary conservation of the binding sites, and the top 25% of interactions ranked by clipExpNum (number of supporting CLIP-seq experiments) to prioritize findings with robust experimental evidence. The resulting miRNA-hub gene regulatory network was visualized using Cytoscape software (version 3.9.1).

### Drug repurposing using the connectivity map

Drug repurposing is a powerful strategy for identifying new therapeutic applications of existing drugs. We used the Connectivity Map (CMap; https://clue.io/) database and its L1000 analysis platform to predict molecularly targeted drugs capable of reversing the gene expression signatures of ASD and IBS. To prioritize drugs with broader therapeutic potential, we identified the compounds common to both ASD and IBS predictions and computed a cumulative normalized connectivity score (norm_cs) for each. A negative connectivity score indicated that the corresponding drug counteracted the disease-associated gene expression pattern.

### Ethics statement

Not applicable.

## Results

### Identification of GRI-associated co-expression modules

**Fig 1:**
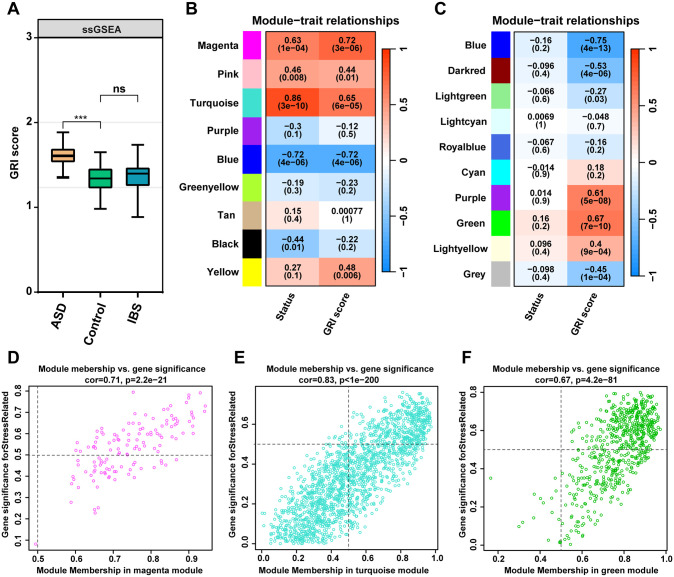
Results of single-sample gene set enrichment analysis (ssGSEA) and weighted gene co-expression network analysis (WGCNA). (A) ssGSEA-based enrichment analysis of glucocorticoid-responsive immune (GRI) genes in autism spectrum disorder (ASD) irritable bowel syndrome (IBS) datasets (***p < 0.001; ns, not significant). (B–C) WGCNA identifying disease- and GRI-associated modules in ASD and IBS, respectively. (D–F) Scatterplots of module membership (MM) versus gene significance (GS) for GRI-associated modules in ASD (Magenta and Turquoise) and IBS (Green). Hub genes were defined as those with |MM| > 0.5 and |GS| > 0.5.

To elucidate the role of GRI genes in the shared molecular architecture of ASD and IBS, we first performed differential expression analysis using the GSE124549 dataset (S1A and S1B Fig). This dataset was significantly enriched for GRI genes, immune cell type–specific transcripts, microRNA-processing factors, and putative stress-related biomarkers [36]. Using a cutoff of *p* < 0.05, we identified 37 differentially expressed genes that were subsequently used as a custom gene set for ssGSEA. As shown in Fig 1A, the GRI ssGSEA enrichment score (GRI score) was markedly elevated in ASD samples compared to controls (*p* < 0.001), indicating enhanced dysregulation of GRI signaling. However, in IBS, the GRI score showed an upward trend without reaching statistical significance, which may reflect the relatively modest systemic immune perturbation and transcriptional divergence between IBS and control samples in this dataset.

To further characterize the transcriptional organization of GRI-related genes, we constructed a WGCNA to identify modules associated with disease status and GRI scores (Fig 1B and C). In ASD, the Magenta and Turquoise modules were positively correlated with both disease status and GRI score (r > 0.5), whereas the blue module showed a negative correlation (r < –0.5). In IBS, the Green module was positively correlated with the GRI score (r > 0.5) and was most strongly associated with disease status, whereas the blue module was negatively correlated (r < –0.5). These consistent patterns suggest a shared co-expression architecture linking GRI activation to disease manifestation in both disorders. To identify robust GRI-associated hub genes, we conducted MM and GS analyses in the ASD Magenta and Turquoise modules and the IBS Green module, applying |MM| > 0.5 and |GS| > 0.5 as selection criteria (Fig 1D and F). This yielded 1,603 candidate genes in ASD and 847 in IBS, with 121 overlapping genes (S1 Table). These genes constitute a preliminary GRI signature that may underlie the shared glucocorticoid-related immune dysregulation in ASD and IBS, serving as a foundation for downstream mechanistic and pharmacological exploration.

### Shared transcriptional alterations and their correlations with the GRI score

**Fig 2:**
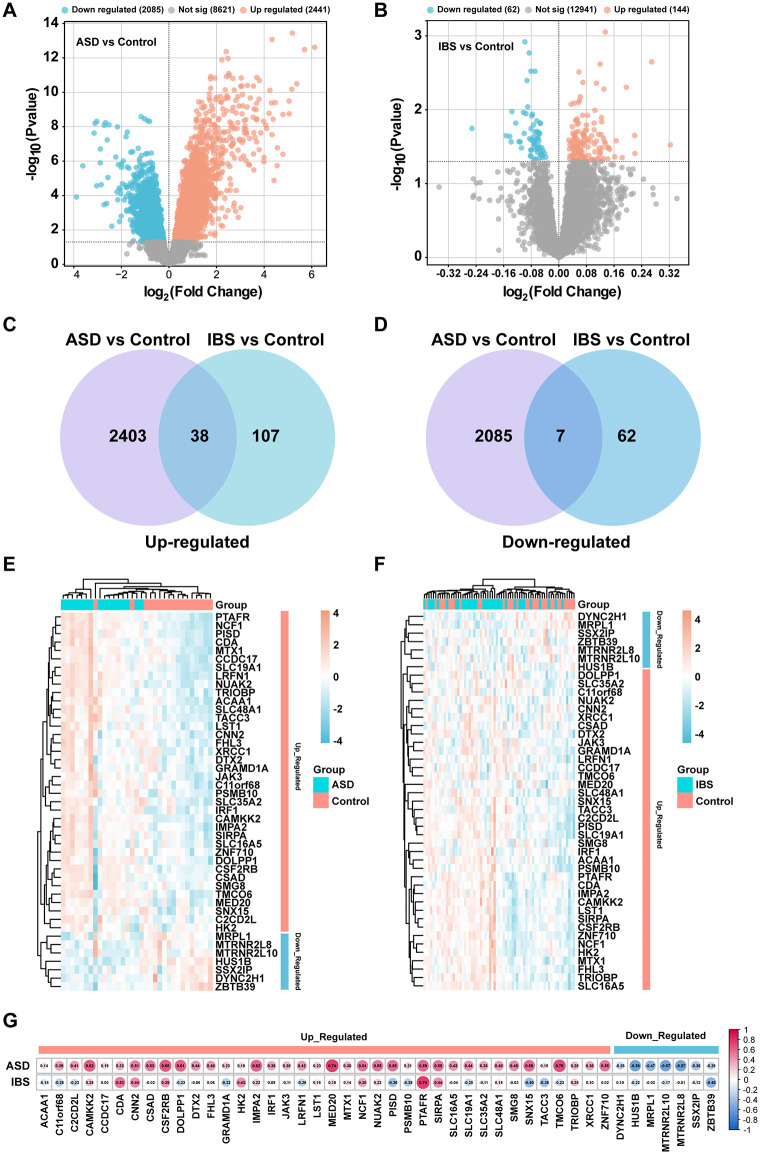
Identification of common differentially expressed genes (DEGs) in autism spectrum disorder (ASD) and irritable bowel syndrome (IBS), and their correlations with glucocorticoid-responsive immune (GRI) score. (A-B) Volcano plots displaying DEGs in ASD and IBS. (C-D) Venn diagrams showing the intersection of up- and down-regulated DEGs between ASD and IBS, respectively. (E-F) Heatmaps of z-score–normalized expression of the shared DEGs. (G) Pearson correlation analyses between shared DEGs and GRI scores in ASD and IBS.

To further refine the GRI signature and explore its underlying biological implications, we conducted differential expression analyses on bulk transcriptomic datasets from both ASD and IBS cohorts (Fig 2A and B). In ASD, 2441 genes were upregulated and, 2085 were downregulated, whereas IBS exhibited 144 upregulated and 62 downregulated genes. Intersecting the upregulated and downregulated gene sets between the two disorders identified 38 commonly upregulated and 7 commonly downregulated genes (Fig 2C and D). Heatmaps of z-score normalized expression values (Fig 2E and F) showed that these shared DEGs robustly discriminated ASD from controls and moderately separated IBS from controls, consistent with disease-

Specific expression intensities. To clarify the relationship between these common DEGs and GRI scores, we performed Pearson’s correlation analyses. In ASD, the shared upregulated genes correlated positively and strongly with the GRI score, whereas downregulated genes showed the opposite pattern. In IBS, correlations were weaker and less uniform, yet the general directionality mirrored that observed in ASD. Together, these findings indicate that the GRI score is closely linked to disease-associated transcriptional alterations, supporting its broader biological relevance and providing additional insights into the molecular commonalities and distinctions between ASD and IBS.

### Network and functional pathway characterization of GRI-associated genes

**Fig 3:**
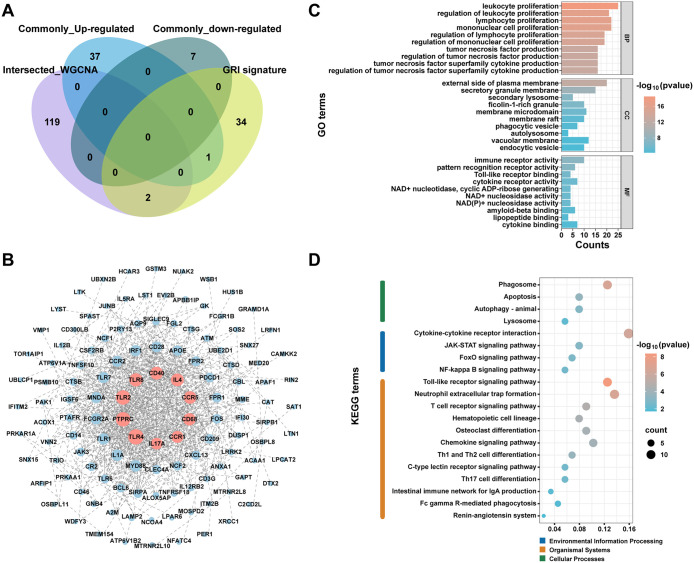
Network and functional characterization of glucocorticoid-responsive immune (GRI)-associated genes. (A) Venn diagram illustrating the integration of the gene sets. (B) Protein-protein interaction (PPI) network of the 125 genes with predicted interaction evidence. (C-D) Gene Ontology (GO) and Kyoto Encyclopedia of Genes and Genomes (KEGG) pathway enrichment analysis. Enriched terms were sorted within their respective categories by descending -log_10_(p-value) to highlight the most statistically significant ones.

To delineate the functional landscape of GRI-associated genes, we integrated the GRI gene set with the intersecting WGCNA hub genes and the common up- and downregulated transcripts shared by ASD and IBS (Fig 3A). After removing duplicates, 200 genes were obtained, of which 125 with protein–protein interaction evidence were visualized using Cytoscape (Fig 3B). Network analysis identified TLR4, PTPRC, TLR2, TLR8, CD40, IL4, CCR5, CD68, and CCR1 as central nodes (ranked in the top 10 degrees), highlighting the prominence of innate immune and cytokine signaling components within the GRI network. Functional GO annotation identified the predominant roles of these genes in leukocyte proliferation, TNF-related biological processes, and immune receptor activity. Concurrently, KEGG pathway analysis showed significant enrichment for key immune signaling pathways (e.g., Toll-like receptor, T-cell receptor, JAK-STAT, and FoxO) and critical cellular processes (e.g., phagosome, apoptosis, and autophagy) (Fig 3C and D). Collectively, these findings indicate that GRI-related transcriptional alterations converge on immune activation and stress-adaptive signaling, providing a mechanistic link between immune dysregulation in ASD and IBS patients.

### Machine learning framework for identifying core GRI-associated drivers

**Fig 4:**
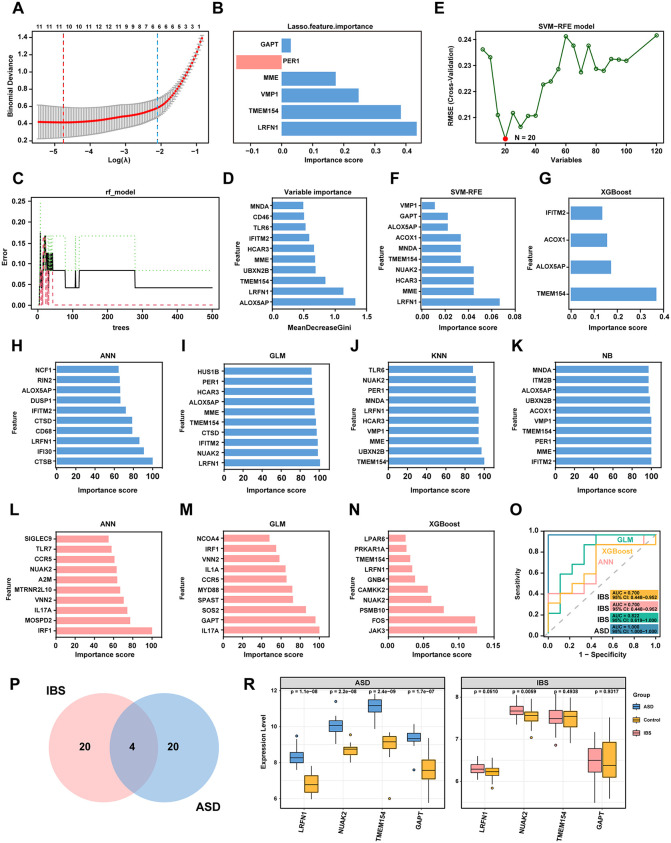
Machine learning-based feature selection from shared glucocorticoid-responsive immune (GRI)-associated genes. (A-N) Performance evaluation and feature importance of eight machine learning algorithms in classifying (A-K) autism spectrum disorder (ASD) and (L-N) irritable bowel syndrome (IBS) samples based on the 125 GRI-associated genes. (O) Receiver Operating Characteristic (ROC) curves with the Area Under the Curve (AUC) value indicating discriminative power (AUC ≥ 0.7). (P) Venn diagram identifying the four shared core candidate genes. (Q) Validation of the expression levels of the four core candidate genes in ASD and IBS bulk transcriptomes. Data are presented with p-values.

To identify the most critical GRI-associated molecules that potentially drive the shared pathology of ASD and IBS, we systematically applied eight machine learning algorithms to the transcriptomic datasets from both disorders (Fig 4A–K for ASD; Fig 4L–N for IBS). Model performance was evaluated based on the ability to distinguish between disease and control samples. In ASD, the GRI gene set demonstrated excellent discriminative power across all models, with an AUC of 1. Although this may partly reflect the limited sample size and representativeness, the consistently high performance underscores the strong disease relevance of GRI-associated transcriptional programs and provides a reliable basis for feature ranking. In contrast, GRI-associated expression patterns in IBS exhibited a more modest classification performance, with three models achieving AUC ≥ 0.7, and the GLM performed best (AUC = 0.822). Given that biologically meaningful features are more likely to emerge from well-performing models, we selected the top ten ranked features from all ASD models and from the three IBS models with AUC ≥ 0.7. The union within each disorder yielded 24 high-importance features, and intersections across disorders identified four shared core candidates: LRFN1, NUAK2, TMEM154, and GAPT (Fig 4P).

We next examined the expression of these four genes in both the ASD and IBS bulk transcriptomes (Fig 4R). All four genes were significantly upregulated in ASD, whereas in IBS, NUAK2 showed significant elevation and LRFN1 displayed a trend toward higher expression (p = 0.051); TMEM154 and GAPT did not differ significantly. These findings suggest that while ASD and IBS share overlapping GRI-related molecular perturbations, the magnitude and coherence of their transcriptional dysregulation differ, with ASD exhibiting a globally disrupted GRI program. Collectively, these analyses highlight LRFN1, NUAK2, TMEM154, and GAPT as putative core regulators of maladaptive GRI signaling, representing promising targets for mechanistic investigations and potential cross-disorder biomarker development.

### Single-cell landscape reveals cell-type-specific perturbations of GRI-associated core genes in ASD

**Fig 5:**
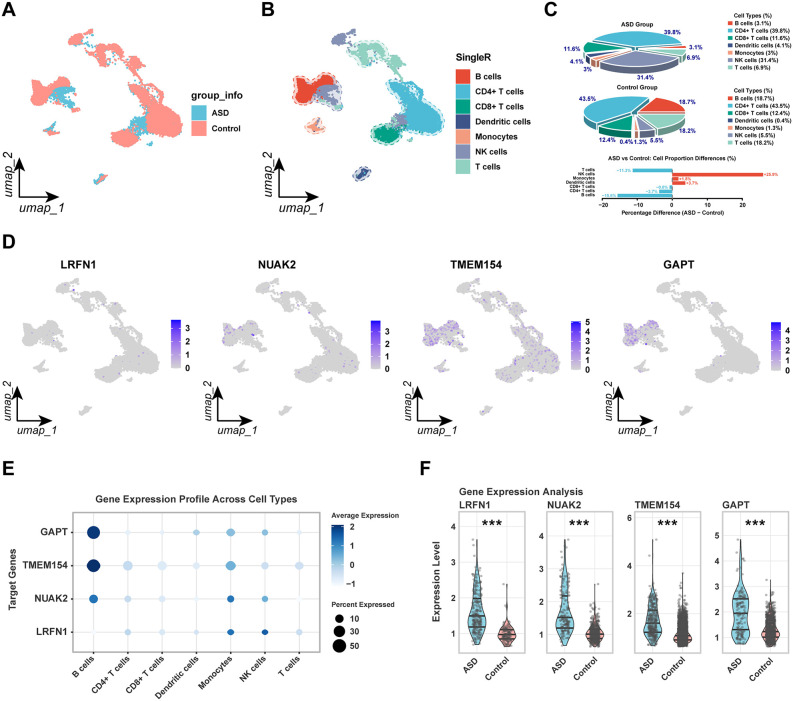
Single-cell RNA analysis. (A) Uniform Manifold Approximation and Projection (UMAP) plot of peripheral blood mononuclear cells (PBMCs) from autism spectrum disorder (ASD) and control samples after batch correction. (B) UMAP plot annotated with major immune cell types. (C) 3-dimensional pie chart comparing the composition of major immune cell populations between ASD and control samples. A bar plot represents percentage point differences in cell type proportions. (D) UMAP plots and (E) dot plot displaying the cell type-specific expression patterns of the four core glucocorticoid-responsive immune (GRI)-associated genes. (F) Violin plots comparing the single-cell expression levels of the four core genes between ASD and control groups across all PBMCs (***p < 0.001).

To further elucidate the cellular context of the identified GRI-associated drivers and their potential immunological relevance in ASD, we performed a single-cell transcriptomic analysis of PBMCs from ASD and control samples. After batch correction, integrated UMAP visualization revealed well-mixed cellular distributions across groups (Fig 5A). Cell type annotation using the *SingleR* algorithm identified the major immune lineages, including B cells, CD4 + T cells, CD8 + T cells, dendritic cells, monocytes, NK cells, and T cells (Fig 5B). A comparison of cell type proportions showed that ASD samples were enriched for NK cells (+25.9%), dendritic cells (+3.7%), and monocytes (+1.8%), whereas B cells (–15.6%), total T cells (–11.3%), and both CD4+ and CD8 + T cells (−3.7% and −0.8%) were reduced relative to controls (Fig 5C). Expression mapping across immune cell subsets revealed distinct cellular preferences for the four GRI-associated core genes. GAPT and TMEM154 were primarily expressed in B cells, NUAK2 was enriched in both B cells and monocytes, and LRFN1 was highly expressed in monocytes and NK cells (Fig 5D and 5E). At the single-cell level, all four genes were significantly upregulated in ASD compared to controls (Fig 5F), consistent with the bulk transcriptomic findings. These indicated that GRI-associated transcriptional dysregulation in ASD is driven predominantly by innate and adaptive immune compartments, particularly B cells, monocytes, and NK cells, supporting an immune-centered mechanism linking GRI signaling to ASD pathophysiology.

### Regulatory network and drug repurposing analysis

**Fig 6:**
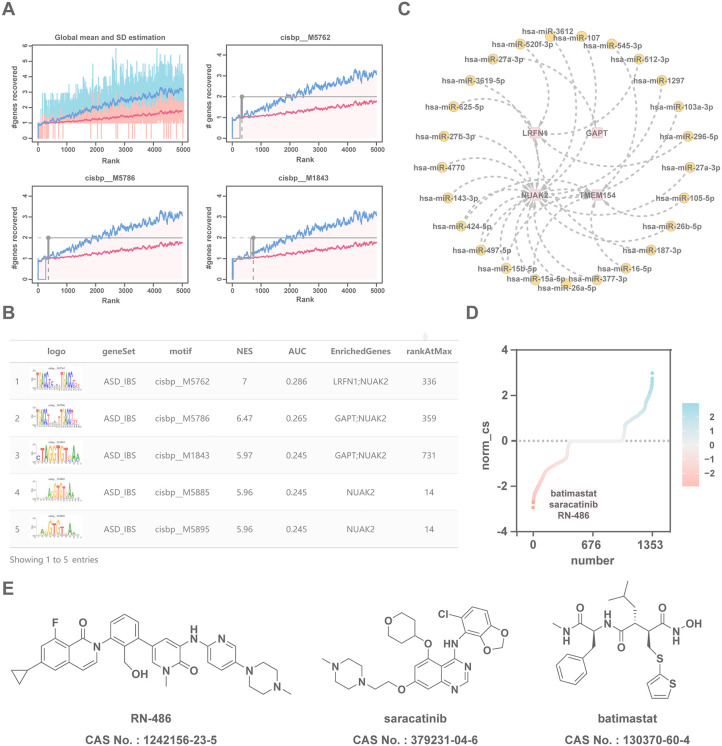
Regulatory network and drug repurposing analysis of glucocorticoid-responsive immune (GRI)-associated core genes. (A–B) Transcription factor (TF) motif enrichment analysis of the four GRI-associated core genes. The plots show the cumulative recovery curve, motif–TF annotation, and enrichment results. (C) Validated miRNA–target interaction network for the four core genes. (D) Connectivity Map (CMap) analysis identifying compounds predicted to reverse GRI-associated transcriptional signatures shared by ASD and IBS. (E) Chemical structures of the top three candidate compounds.

To further elucidate the up- and downstream regulatory mechanisms of the four GRI- associated core genes, we first performed TF motif enrichment analysis (Fig 6A and 6B). Top three enriched motifs ranked by NES were cisbp__M5762 (NES = 7.00), cisbp__M5786 (NES = 6.47), and cisbp__M1843 (NES = 5.97), which were predominantly associated with LRFN1, NUAK2, and GAPT, respectively. These findings suggest that the transcriptional regulation of GRI core genes may converge on shared upstream TF networks involved in immune signaling. In parallel, we conducted miRNA target prediction for these four genes (Fig 6C). Several validated miRNAs were identified, indicating that these genes are likely subject to extensive post-transcriptional regulation, potentially serving as key nodes in miRNA-mediated regulatory circuits. Finally, we employed CMap analysis to identify compounds capable of reversing the transcriptional signatures of the GRI-associated genes shared by ASD and IBS. The top three candidate compounds were RN-486, saracatinib, and batimastat (Fig 6D and 6E). Notably, RN-486 is a BTK inhibitor, saracatinib is an Src family kinase inhibitor, and batimastat is a broad-spectrum matrix metalloproteinase (MMP) inhibitor, collectively implying that the inhibition of BTK, Src, and MMP signaling may mitigate GRI-associated transcriptional dysregulation.

Together, these results reveal a multilayered regulatory framework of GRI-associated genes, integrating transcriptional, post-transcriptional, and pharmacological dimensions that may jointly orchestrate immune responses in ASD and IBS.

## Discussion

The high prevalence of co-occurring GI conditions, particularly IBS, in individuals with ASD represents a significant clinical challenge and points to a shared systemic pathophysiology [5,37]. However, the substantial variation in global ASD prevalence, ranging from approximately 0.01% to 4.36%, suggests that the disorder encompasses a vast clinical spectrum with widely varying symptom severity and diagnostic contexts. Such inherent heterogeneity underscores the urgent necessity of establishing objective biological markers that transcend diverse demographic and diagnostic backgrounds [2]. In this study, we preliminarily delineated a unified molecular framework that links GRI dysregulation to the overlapping pathophysiology of ASD and IBS. Through integrated bulk and single-cell transcriptomic analyses combined with machine learning and network modeling, we revealed that perturbations in GRI signaling in PBMCs potentially form a shared biological axis connecting neurodevelopmental and gut-brain inflammatory processes.

At the transcriptional level, GRI-associated gene expression patterns robustly distinguished ASD from, to a lesser extent, IBS in controls. This discrepancy underscores a critical nuance; the nature of GRI dysregulation may differ between the two conditions. ASD may be characterized by a more tonic, globally dysregulated state, whereas IBS might be a phasic phenomenon, exacerbated during periods of stress or symptom flare-ups, which could be diluted in “cross-sectional” analyses [38]. Further network and functional analyses reveal that the GO annotations highlighting “PBMC proliferation” and “TNF-related biological processes” are tightly coupled to the signaling networks identified in our KEGG enrichment and protein-protein interaction results. Specifically, the activation of Toll-like receptor and T-cell receptor signaling, highlighted by central nodes such as TLR4 and PTPRC (CD45), serves as a common trigger in both disorders, initiating a pro-inflammatory cascade. This cascade is powerfully amplified through the production of TNF, which subsequently drives aberrant proliferation and activation of leukocytes [39]. Recent evidence also indicates dysregulated TNF signaling within the circulating immune cells of individuals with ASD [40]. The JAK–STAT cascade functions as a crucial relay, transmitting cytokine-derived signals to the nucleus to activate transcriptional programs that control immune cell growth and differentiation [41]. In parallel, the FoxO, apoptosis, and autophagy pathways integrate immune and metabolic cues to maintain homeostasis by regulating cell survival and turnover. Together, these define the pathophysiological outputs of an interconnected immune signaling network, providing a mechanistic explanation for the chronic immune activation that is common to ASD and IBS.

To further identify the GRI-associated core genes, we applied a machine learning-based framework, revealing four core candidates—LRFN1, NUAK2, TMEM154, and GAPT—as pivotal mediators of neuroimmune crosstalk in ASD and IBS. These genes, which are overexpressed in ASD and variably dysregulated in IBS, suggest that shared genetic vulnerabilities are shaped by both central and peripheral stressors. LRFN1 is a neuronal synaptic adhesion molecule critical for excitatory synapse formation and N-methyl-D-aspartate (NMDA) receptor stabilization [42]. Notably, LRFN1 has been directly linked to ASD through the identification of a de novo heterozygous missense variant in a child with non-syndromic ASD, implying its potential role as an ASD-associated synaptopathy gene [43]. In the context of PBMC, the presence of LRFN1 highlights a potential extraneuronal function in regulating immune cell communication and motility, positioning it as a plausible molecular bridge that links neuroinflammatory activity to peripheral immune responses. The AMPK-related kinase NUAK2, activated under metabolic stress, has been shown to enhance GRI signaling in immune cells [44]. Beyond its established role in cell proliferation through the TNF-α/NF-κB/NUAK2/LATS/YAP axis, recent evidence has demonstrated that the susceptibility gene [45,46]. Collectively with the known ASD association of NUAK2 mutations, these findings suggest that NUAK2 may bridge neurodevelopmental and intestinal inflammatory processes, highlighting its potential relevance to both ASD and IBS. On the other hand, functional annotation of TMEM154 is limited, with current evidence primarily describing its involvement in viral binding and host susceptibility [47,48]. Overexpression of TMEM154 in PBMCs may increase cellular vulnerability to viral infection or immune dysregulation, thereby exacerbating systemic stress and the inflammatory burden in individuals with these disorders. Moreover, a recent *Cell* study identified TMEM154 (Galvanin) as an electric-field sensor that directs immune/epithelial cell migration, suggesting a broader role in inflammation and immune trafficking [49], although direct links to ASD or IBS remain unestablished. Lastly, GAPT functions as a negative regulator of B cell activation, and no direct associations with ASD or IBS have been reported [50]. Its overexpression likely reflects a compensatory adaptation to chronic immune stimulation, contributing to the maladaptive immune response observed in ASD, a pattern corroborated by scRNA sequencing analysis. Single-cell profiling revealed a shift in the peripheral immune landscape of ASD patients toward heightened innate immune activation and impaired adaptive immune regulation. Specifically, the increased abundance of NK cells, dendritic cells, and monocytes reflects a proinflammatory milieu indicative of chronic immune activation and HPA axis dysregulation, consistent with elevated circulating cytokines, such as IL-1β, IL-6, and TNF-α, reported in ASD cohorts [51]. Conversely, the marked reduction in total T cells, including both CD4 + and CD8 + subsets, along with decreased B cell proportions, suggests GRI-associated suppression of lymphocyte proliferation and breakdown of immune tolerance [52,53]. Together, the malfunction of LRFN1, NUAK2, TMEM154, and GAPT underscores the multifaceted disruption of PBMC homeostasis, which manifests as aberrant cell turnover, heightened immune sensitivity, and altered immune cell distribution, which may collectively reinforce systemic inflammation and neuroimmune dysfunction in ASD and IBS.

A critical consideration in interpreting the GRI signature is the potential confounding influence of anxiety and depression, which frequently co-occur with both ASD and IBS [54]. From a mechanistic perspective, these psychiatric comorbidities often share a common pathological substrate with the gut–brain axis, namely HPA-axis hyperactivation and systemic low-grade inflammation. It is therefore possible that certain identified candidates, such as NUAK2 or GAPT, partially reflect generalized stress-responsive pathways rather than an exclusive link between ASD and IBS. However, the presence of genes like LRFN1, a synaptic adhesion molecule more closely associated with neurodevelopmental synaptopathy than with transient stress-induced plasticity, suggests that the GRI framework maintains a degree of specificity [43], rather than simple stress-induced plasticity. This implies that while the molecular landscapes of ASD, IBS, and affective disorders may be intertwined, the GRI signature likely captures a core neuroimmune crosstalk that persists across diverse, medication-free cohorts. Rather than viewing these psychiatric symptoms as mere noise, they may be seen as synergistic components of a broader dysregulation within the neuroimmune-endocrine axis, the specific drivers of which warrant further investigation in more clinically granular studies.

Upstream regulatory and drug perturbation analyses have shed light on the control hierarchy governing these molecular alterations. Motif enrichment implicated CIS-BP transcription factors, particularly cisbp _ M5762, cisbp__M5786, and cisbp__M1843, as potential upstream regulators of LRFN1, NUAK2, and GAPT, aligning with stress- and inflammation-sensitive transcriptional networks. Extensive miRNA targeting predicted for these genes suggests a complex post-transcriptional regulation that reinforces immune adaptability. CMap analysis identified RN-486, saracatinib, and batimastat as top candidate compounds capable of reversing the shared ASD–IBS GRI signature. These candidates offer a compelling mechanistic rationale for therapeutic intervention across the gut–brain–immune axis. Specifically, RN-486 acts as a potent and selective inhibitor of BTK, which effectively suppresses pro-inflammatory cascades (e.g., IL-6 and IL-8) and mast cell degranulation—key drivers of intestinal and systemic inflammation. Saracatinib targets the Src family kinases (including Lck, Lyn, and Fyn), providing potential benefits in stabilizing synaptic plasticity and modulating neuroinflammatory responses. Furthermore, batimastat serves as a broad-spectrum MMP inhibitor that regulates extracellular matrix remodeling, a process essential for maintaining tissue barrier integrity. By targeting these convergent pathways of immune-mediated inflammation and structural homeostasis, these compounds represent promising strategies for restoring GRI equilibrium [55–57]. However, their translational potential for treating ASD-IBS requires further experimental validation using relevant biological models.

While this study provides a robust computational framework for the GRI-mediated link between ASD and IBS, empirical validation remains essential to translate these bioinformatic insights into clinical relevance. Future research should prioritize a multi-tiered experimental strategy encompassing *in vitro*, *in vivo*, and clinical validation. First, at the cellular level, patient-derived PBMCs from age- and sex-matched cohorts should be utilized to validate the expression levels of the identified core biomarkers—LRFN1, NUAK2, TMEM154, and GAPT—via qPCR and Western blot. To verify the functional impairment of GC responsiveness, dexamethasone suppression tests on cultured immune cells can be performed to observe whether these markers exhibit the predicted resistance or hypersensitivity to corticosteroid stimulation. Second, in vivo mechanistic studies are warranted to explore the causal relationship within the gut–brain–immune axis using mouse models of ASD. Common models include genetic knockouts (e.g., *Shank3*, *Cntnap2*) and inbred strains like BTBR, which are used to study pathophysiology and test potential therapies [58]. Utilizing transgenic mouse models or AAV-mediated localized knockdown of LRFN1 or NUAK2 would allow researchers to assess key physiological indicators, such as intestinal epithelial permeability and neuroinflammatory markers, including microglial activation and cytokine profiles in the prefrontal cortex and hippocampus. Such studies would clarify whether GRI dysregulation serves as a primary mechanical driver of the observed neuro-gastrointestinal overlap. Finally, the pharmacological candidates identified through our CMap analysis, such as RN-486 or other target-specific inhibitors, should be evaluated in preclinical “rescue” experiments to determine their efficacy in reversing both behavioral abnormalities and GI dysmotility. These integrated experimental designs will be crucial for disentangling the complex interplay between the HPA axis, systemic immunity, and the dual-organ pathology of ASD and IBS.

Overall, our findings suggest that dysregulated signaling within PBMCs shared by ASD and IBS patients could be represented by a customized GRI signature, and that pharmacological inhibition of these pathways could potentially restore peripheral immune homeostasis and mitigate neuroimmune dysregulation.

### Limitations and future directions

This study had several limitations. First, although we employed a parallel analysis framework and internally matched disease cases and controls for age and sex, the inherent heterogeneity and batch effects of public transcriptomic datasets may still influence the results and model robustness. Specifically, although available metadata were meticulously audited, some clinical variables, such as medication history and inflammatory status, were not documented in all repositories. Furthermore, common psychiatric comorbidities such as anxiety and depression, along with HPA-axis dysregulation, may act as confounders that are difficult to isolate in public metadata; thus, our GRI signature might partially reflect these shared stress-related pathways, necessitating future validation with more granular clinical profiling. Second, despite implementing a stringent train–test split and cross-validation strategy, the relatively modest sample size and the absence of an independent external validation cohort may limit the generalizability of the machine learning findings. Third, the study is correlative in nature; experimental validation of GRI-associated gene function in immune and neuronal contexts remains essential for establishing causality. Finally, while CMap analysis provides mechanistic hypotheses, pharmacological reversal of the GRI signature requires in vitro and in vivo confirmation. Future studies integrating large-scale multi-omic longitudinal datasets, glucocorticoid challenge experiments, and cell-type-resolved perturbation assays will be critical to validate and expand these findings. Ultimately, translating GRI modulation into therapeutic benefits will require a deeper understanding of how systemic stress signaling coordinates neuroimmune homeostasis across the brain and gut axes.

## Conclusion

In summary, our integrative multi-omic and computational analyses identified dysregulated GRI signaling as a convergent molecular axis linking ASD and IBS. Through transcriptomic profiling, network inference, and machine learning prioritization, we identified a core set of GRI-associated genes—LRFN1, NUAK2, TMEM154, and GAPT—that bridge neuroimmune and gut inflammatory pathways. Single-cell resolution further demonstrated that these genes are predominantly expressed in innate immune populations, implicating maladaptive stress–immune interactions in disease pathophysiology. Upstream transcriptional and post-transcriptional analyses, together with drug perturbation modeling, suggest that targeting kinase- and matrix-related pathways may restore glucocorticoid sensitivity and immune equilibrium.

Overall, these findings re-emerge as ASD and IBS as interconnected disorders of systemic stress adaptation rather than isolated neurological or GI conditions. By elucidating the shared GRI-centered regulatory architecture, this work provides a foundation for developing mechanism-based interventions aimed at normalizing neuroimmune crosstalk along the brain–gut axis.

## Supporting information

S1 Table Intersection of WGCNA-derived hub genes between ASD and IBS associated with the GRI score. (XLSX)

S1 Fig Differential expression analysis of GSE124549 dataset. (A) Volcano plot of differentially expressed genes (DEGs); (B) Heatmap of DEGs, clustered by samples and expression levels.
(TIF)
