# Supplementary figures and images for: Dysregulated glucocorticoid-responsive immune genes in peripheral blood mononuclear cells as a shared molecular signature of autism spectrum disorder and irritable bowel syndrome
Source: PLoS One. 2026 Jul 9;21(7):e0353181. doi: 10.1371/journal.pone.0353181 (PMC13349188; doi:10.1371/journal.pone.0353181)

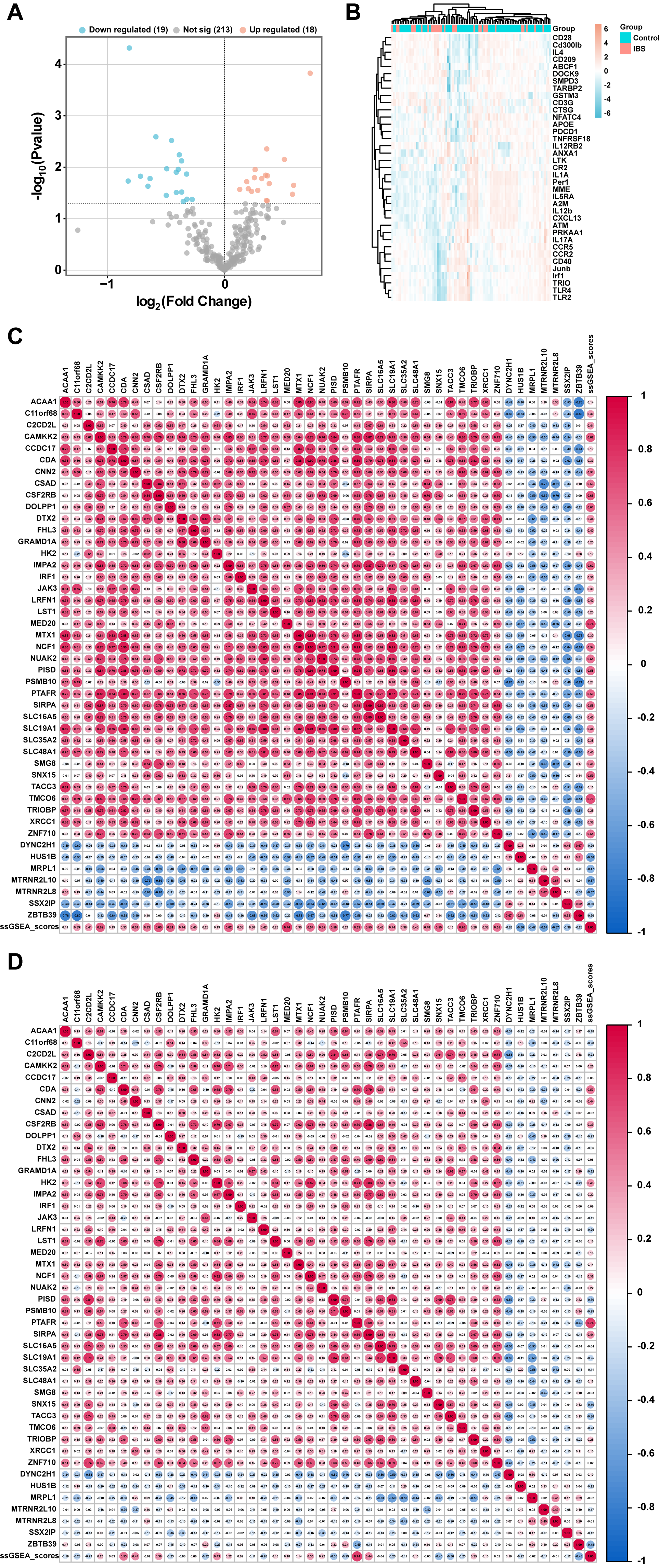

Supplement: S1 Fig — (A) Volcano plot of differentially expressed genes (DEGs); (B) Heatmap of DEGs, clustered by samples and expression levels. (TIF) [file pone.0353181.s002.tif]
